# Changes in circulating microRNA levels associated with prostate cancer

**DOI:** 10.1038/bjc.2011.595

**Published:** 2012-01-12

**Authors:** R J Bryant, T Pawlowski, J W F Catto, G Marsden, R L Vessella, B Rhees, C Kuslich, T Visakorpi, F C Hamdy

**Affiliations:** 1 Nuffield Department of Surgical Sciences, University of Oxford, Oxford, UK; 2 Caris Life Sciences, Research and Development Division, Phoenix, USA; 3 Department of Oncology, University of Sheffield, Sheffield, UK; 4 Department of Urology, University of Washington, Seattle, USA; 5 Institute of Biomedical Technology and BioMediTech, University of Tampere and Tampere University Hospital, Tampere, Finland

**Keywords:** prostate cancer, microRNA expression, serum, plasma, urine

## Abstract

**Background:**

The aim of this study was to investigate the hypothesis that changes in circulating microRNAs (miRs) represent potentially useful biomarkers for the diagnosis, staging and prediction of outcome in prostate cancer.

**Methods:**

Real-time polymerase chain reaction analysis of 742 miRs was performed using plasma-derived circulating microvesicles of 78 prostate cancer patients and 28 normal control individuals to identify differentially quantified miRs.

**Results:**

A total of 12 miRs were differentially quantified in prostate cancer patients compared with controls, including 9 in patients without metastases. In all, 11 miRs were present in significantly greater amounts in prostate cancer patients with metastases compared with those without metastases. The association of miR-141 and miR-375 with metastatic prostate cancer was confirmed using serum-derived exosomes and microvesicles in a separate cohort of patients with recurrent or non-recurrent disease following radical prostatectomy. An analysis of five selected miRs in urine samples found that miR-107 and miR-574-3p were quantified at significantly higher concentrations in the urine of men with prostate cancer compared with controls.

**Conclusion:**

These observations suggest that changes in miR concentration in prostate cancer patients may be identified by analysing various body fluids. Moreover, circulating miRs may be used to diagnose and stage prostate cancer.

Prostate cancer is the most commonly diagnosed male malignancy and the second leading cause of male cancer-related death (Jemal *et al*, 2010). While community prostate-specific antigen (PSA) testing can lead to the early detection and diagnosis of prostate cancer this test has a low specificity, and the optimal threshold for biopsy is unclear (Thompson *et al*, 2003). In addition, PSA screening leads to the over diagnosis and over treatment of indolent prostate cancers (Ciatto *et al*, 2000; Dall’Era *et al*, 2008). In contrast, men receiving radical treatment for presumed locally confined prostate cancer often develop disease relapse post treatment, and in the majority of these patients the disease was more extensive than it appeared pre-treatment.

MicroRNAs (miRs) are short non-coding RNA molecules with an average length of 22 nucleotides (Catto *et al*, 2009). They are transcribed as RNA hairpins and processed into mature miRs that bind to complementary messenger RNA to alter gene expression (Bartel, 2009). Currently around 1000 human miRs have been identified and each of these may target around 1000 genes (Bartel, 2009) leading to a complex layer of control of signalling pathways important to the development or progression of cancers (Croce, 2009). Several miRs have altered function or are differentially present in prostate cancer (Catto *et al*, 2011). These appear to have important mechanistic roles with respect to apoptosis avoidance, proliferation, epithelial-to-mesenchymal transition and development of androgen independence. MicroRNAs may be useful biomarkers as their relatively small size protects them from RNase attack and they are secreted within protective exosomes (Weber *et al*, 2010). Several miRs have been shown to be upregulated in sera of men with prostate cancer compared with normal controls (Mitchell *et al*, 2008; Brase *et al*, 2011); however, to date most of these studies contain small numbers of ill-defined patients.

Current investigations to detect and stage prostate cancer are unable to detect micro-metastases (Rajarubendra *et al*, 2010). Consequently, men with an undetectable low metastatic burden undergo radical therapy for incorrectly presumed localised prostate cancer. Therefore tests with greater staging accuracy are urgently needed to accurately stratify patients. The aim of this study was to perform a high-throughput analysis of a wide range of miRs in serum and plasma samples of men with different stages of prostate cancer and controls in order to identify those associated with the presence and extent of prostate cancer. We also used urinary samples to explore the identified miRs in another body fluid.

## Materials and methods

### Samples

*ProMPT* Plasma samples from prostate cancer cases (*n*=78, 12 not evaluated for metastases, 51 M0, 15 M1) and normal control individuals (*n*=28) were selected from men in the ProMPT (Prostate Cancer Mechanisms of Progression and Treatment) study. For the purposes of this study normal control individuals were men with a PSA of <10 ng ml^−1^ who had undergone one or more negative prostate biopsies. The study had ethics committee approval (UK MREC number 01/4/61). After informed consent, BD PPT K2E plasma samples were collected using vacutainer tubes (Cat # 362799). Within 10 min of blood draw, the blood samples were centrifuged at room temperature using the Capricorn CEP2000 for 20 min at 2200 r.c.f. The samples were then aliquoted and frozen at −80 °C within 24 h.

Urine samples enriched for prostatic cells were collected from 135 men following trans-rectal digital massage as described elsewhere (Hessels *et al*, 2003). The first 5 ml of voided urine post-examination was collected, stored at 4 °C for up to 4 h and then centrifuged at 3392 **g** for 10 min. The supernatant was removed and the cell pellet washed twice in PBS before freezing at −80 °C until use.

*University of Washington* Serum samples were collected from patients following radical prostatectomy who subsequently developed recurrent metastatic prostate cancer (*n*=47) or who subsequently had non-recurrent disease (*n*=72). Serum from non-recurrent prostate cancer patients was negative for PSA in all cases. Patients with non-recurrent prostate cancer were followed up with PSA measurements at 5 months or greater post-surgery. Samples were collected in a serum separator tube and serum was aliquoted in 1 ml aliquots and kept frozen at −80 °C. These samples were from the Genitourinary Cancer Biospecimen Repository from the University of Washington and had appropriate IRB approval (IRB approval number 39053). The samples were transferred to and stored at Caris Life Sciences (Phoenix, AZ, USA) for analysis.

### Sample preparation and RNA extraction

*Plasma samples* Frozen plasma samples were thawed and enriched populations of cMVs were obtained by then filtering the plasma through a 1.2-*μ*m filter to remove cells and cellular debris. One ml of the filtrate was concentrated to 300 *μ*l final retentate volume on a filter concentrator with a 150-kDa molecular weight cutoff. Samples were treated with Rnase A (Ambion, Austin, TX, USA) before RNA extraction in order to ensure that RNA was derived from cMVs. Total RNA from 150 *μ*l of concentrated cMV was extracted using a modified version of the Qiagen miRNeasy extraction protocol and eluted in a final volume of 150 *μ*l. All samples were spiked with 25 fmol *μ*l^−1^ of *Caenorhabditis elegans* miR 39 for use as a normaliser in downstream analyses.

*Serum samples* In order to ensure that the RNA was from cMVs 500 *μ*l of serum was treated with 3 *μ*g ml^−1^ Rnase A. After Rnase A treatment, to ensure that isolated RNA was indeed from cMVs, the RNA was isolated with the ExoMiR extraction kit (Bioo Scientific, Austin, TX, USA), according to the manufacturer's instructions. Samples were spiked with 25 fmol *μ*l^−1^ of *C. elegans* miR-39 for use as a normaliser in downstream analyses. The ExoMir extraction method is designed to isolate RNA from the microvesicle and exosome portions of the serum sample. A clarifying spin was performed in order to remove cellular fragments before the samples were pushed through the filters. The serum samples were pushed through two different sized filters (the first filter catches the larger microvesicles, while the second filter catches the smaller exosomes sized between 20–200 nm) and then the RNA was isolated by eluting the sample off the filters with lysis solution.

*Urine samples* Total RNA was extracted from thawed cell pellets using the mirVana kit (Ambion) according to the manufacturer's protocol and measured using a 2100 Bioanalyzer (Agilent, Cheshire, UK).

### qRT–PCR screening

In total 40 ng of RNA from plasma samples was reverse-transcribed using the miRCURY LNA Universal RT miR PCR, polyadenylation and cDNA synthesis kit. This amount of RNA was chosen in order to ensure that there was enough input material for the Exiqon qRT–PCR microarray panels to work. cDNA was then screened using qRT–PCR on the miR ready-to-use PCR Human Panel I+II (Exiqon Inc., Woburn, MA, USA) on an ABI 7900 Sequence Detection System.

### qRT–PCR verification

Selected miRs were measured using Taqman human miR assays (Life Technologies, Carlsbad, CA, USA). In brief, 3 *μ*l of RNA was reverse transcribed using the multi-scribe reverse transcriptase kit for each assay studied. cDNA was pre-amplified with molecular grade nuclease-free water as a diluent, and the cDNA was then assessed using the Taqman qRT–PCR assay following manufacturer recommendations on an ABI 7900 Sequence detection system. Serum and urine samples were assessed by the same method with the only exception that the initial reverse transcription reaction was carried out with a custom multiplex primer mix specific to the assays being assessed. Absolute quantification was measured by creating a standard curve for each assay.

### Statistical analysis

The miR ready-to-use PCR Human Panels I+II (Exiqon Inc) contain inter-plate as well as reverse transcription calibrators. These were used to normalise the data after it was exported to GeneSpring GX v11 (Agilent, Santa Clara, CA, USA) for analysis. Normalised values for all prostate cancer *vs* controls, non-metastatic prostate cancer *vs* controls and non-metastatic *vs* metastatic analyses were subjected to a fold-change analysis. Those miRs found to have a fold change greater than 2 were then subjected to an unpaired *t*-test. The Benjamini and Hochberg false detection test (Wang *et al*, 2009) was applied to the whole data set.

Taqman verification assays were analysed by first normalising the CT of a selected assay to the *C. elegans* miR-39 Taqman CT for the same sample. This value was multiplied by the copy number for a selected assay to arrive at the corrected copy number. Assay median copy numbers for each group compared were assessed using a Mann–Whitney *U* test. This spike-in method of normalisation was chosen because the frequently used sno normalisers are not expressed in cMVs. For the urinary samples, we normalised miR expression to the mean of two reference snoRNAs (RNU44 and RNU48).

For the multivariate PSA and miR analysis of the Taqman data, we reviewed the original data set for missing data patterns. A subset of *n*=103 subjects (25 control individuals and 78 prostate cancer patients) were identified with complete data for PSA and miRs 107, 130b, 326, 301a, 185, 625 and 2110 (other miRs showed extensive missing data patterns and were excluded). We considered each miR as a possible class differentiator for prostate cancer, and only two miRs (miRs 107 and 326) were significantly associated with prostate cancer at the *P*=0.1 comparison-wise level, suggesting that any association between marker and disease outcome is likely to be weak. In contrast to the miR data, the use of PSA level as a predictor of disease was more robust as it returned a *P*-value <1E-5. For completeness, we assessed the performance of these miR markers with and without PSA using various predictive modeling techniques (e.g., support vector machines, diagonal linear discriminant analysis and logistic regression). Base models (PSA alone) had AUCs generally in the order of 0.75. Models with miRs and AUCs were 0.75–0.80. The results for multivariate models were therefore consistent with univariate tests and demonstrated that miRs did not show evidence either individually or collectively of being able to improve the detection of prostate cancer.

Support vector machines and their corresponding ROC curves and AUC statistics were fit in R 2.12 (R development Core Team, Vienna, Austria, 2011 ISBN 3-900051-07-0) using the CMA package (Slawski *et al*, 2008).

## Results

### Patients and samples

**Figure 1:**
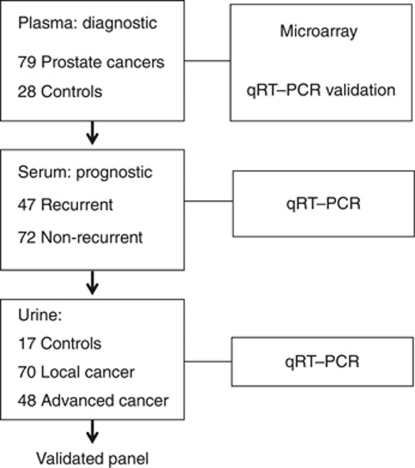
Overview of miR analysis of plasma, serum and urine samples.

The demographics and clinical characteristics of the 106 ProMPT plasma sample patients, 135 ProMPT urine sample patients and 119 University of Washington serum sample patients are outlined in Table 1. An overview of the miR analysis of plasma, serum and urine samples outlined in this manuscript is presented in Figure 1.

### qRT–PCR array identification of miRs associated with prostate cancer

First, we performed an analysis of plasma-derived cMVs in the 78 cancer cases and 28 controls within the ProMPT study, using an Exiqon qRT–PCR microarray panel of 742 miRs. A total of 12 miRs were differentially quantified, 11 were significantly increased in the plasma-derived cMVs of prostate cancer patients compared with normal control individuals, while the concentration of miR-181a-2^*^ was significantly decreased (*P*<0.05 unpaired *t*-test) (Table 2A). We then analysed plasma-derived cMVs from 55 non-metastatic prostate cancer patients and 28 normal control individuals to identify miR changes associated with the presence of non-metastatic disease. In all, 10 miRs were found to be differentially quantified, 9 miRs showed a significant increase, while miR-181a-2^*^ showed significant decrease in the plasma cMVs of prostate cancer patients without distant metastases compared with normal control individuals (*P*<0.01 unpaired *t*-test) (Table 2B). We then compared the miR profiles of 16 patients with metastatic and 55 patients with non-metastatic prostate cancer. A total of 16 miRs were found to be differentially quantified in prostate cancer patients with metastases compared with those with non-metastatic disease. A total of 15 miRs showed significantly greater concentration, whereas miR-572 was significantly less (*P*<0.01 unpaired *t*-test) (Table 2C) in men with metastatic *vs* non-metastatic prostate cancer.

### Multivariate analysis of PSA and miRs

Multivariate analysis of the PSA values and the miR levels showed that neither measure was superior in predicting prostate cancer. In this cohort of non-metastatic prostate cancer patients compared with biopsy confirmed normal individuals, PSA had an AUC of 0.79 with a sensitivity of 75% and a specificity of 46% at 4 ng *μ*l^−1^. In contrast, miR-107 had a sensitivity of 67% and a specificity of 43% at 3000 copies and an AUC of 0.62. Combining PSA and miR-107 and 141 did not enhance the performance of either assay in detecting prostate cancer.

In the metastatic *vs* non-metastatic prostate cancer cohort, 77 samples characterised as either M0 or M1 were assayed by individual Taqman qRT–PCR for five miR markers (miRs 375, 107, 200b, 141 and 452) found to have either significant or non-significant expression differences in the initial Exiqon qRT–PCR microarray panel. Of these markers miR 375 and 141 were individually significantly associated with metastatic disease (*P*<0.01) by unequal variance *t*-test. To estimate the combined predictive value of these two miRs, with and without PSA, we formed support vector machine classification models using a linear kernel. Both methods performed well (estimated AUCs of 0.80 for each), suggesting the potential predictive value of a miR-based test. However, given the limited sample size of this data set it was not possible to determine whether the combination of miR and PSA measurement improved upon the model using miR alone.

### qRT–PCR confirmation of miRs associated with prostate cancer

Taqman qRT–PCR assays were performed in order to validate the Exiqon qRT–PCR microarray panel miR quantification changes identified in our ProMPT plasma samples. MicroRNA-107 and miR-574-3p were increased in cMVs of men with non-metastatic prostate cancer compared with normal control individuals (*P*<0.05 Mann–Whitney *U* test). MicroRNA-141, miR-375 and miR-200b were differentially quantified in men with metastatic prostate cancer compared with individuals with non-metastatic disease (*P*<0.05 Mann–Whitney *U* test). MicroRNA-375 and miR-141 concentrations were found to be highly correlated with an *r*^2^=0.55 (Spearman correlation test *P*<0.001).

### Confirmation of the metastatic miR signature

**Figure 2:**
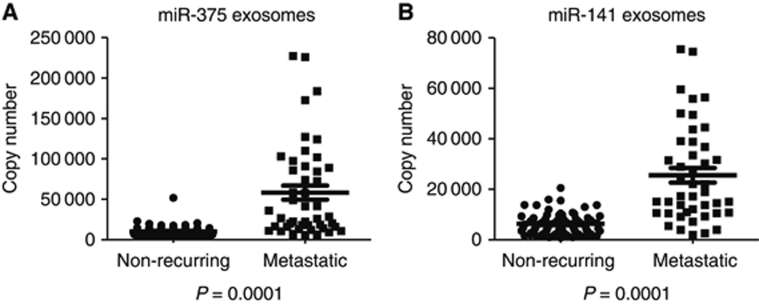
Taqman qRT–PCR analysis using an independent University of Washington serum cohort of exosome fractions verified the quantification changes of miR-375 (**A**) and miR-141 **(B)** (*P*=0.0001 Mann–Whitney *U* test).

In order to confirm the metastatic miR signature identified in the ProMPT plasma samples, we performed an analysis of an independent serum cohort from the University of Washington. This cohort consists of separate microvesicle and exosome portions taken from sera from 47 patients with prostate cancer metastases and 72 patients with non-recurrent prostate cancer. We were interested in examining both the MV and exosome portion separately, as the plasma concentration method used to gather the cMVs collects both portions, and we wished to see if there was a difference in miR quantification in these two blood fractions. This analysis confirmed that miR-375 and miR-141 are significantly increased in both the exosome (Figure 2) and microvesicle (data not shown) portions of sera from patients with metastatic prostate cancer compared with non-recurrent prostate cancer patients (*P*=0.0001 Mann–Whitney *U* test).

### Urinary miR expression

**Figure 3:**
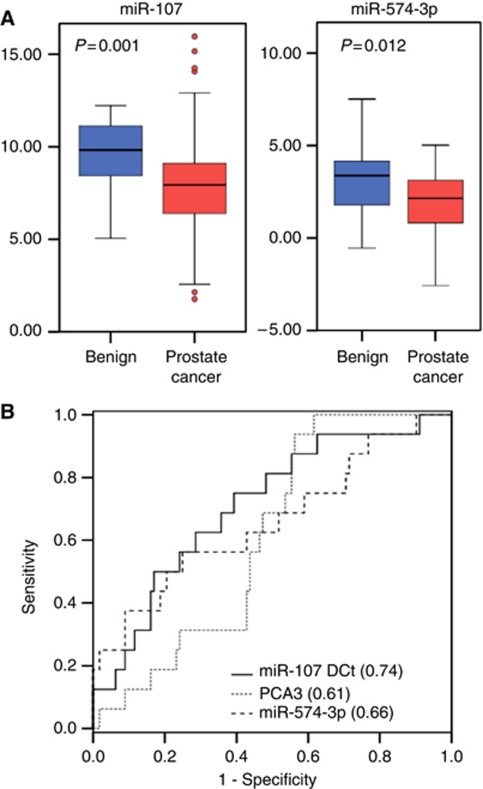
MicroRNAs-107 and 574-3p in urinary prostate cells are associated with the presence of cancer. (**A**) The quantity (shown as ΔCt values with respect to reference snoRNAs) is higher in prostate cancer cases, when compared with controls. (**B**) This quantification can be used to identify the presence of prostate cancer from urine samples (AUC ROC shown in brackets).

Having observed numerous miR expression changes in the plasma and serum of prostate cancer patients compared with normal control individuals, we next investigated the possibility that some of these miR changes might also be found in another fluid sample that had been obtained from our cohort of men. We successfully quantified the concentration of five selected miRs in 135 samples. When RNA values normalised to the mean of RNU44/RNU48 (endogenous controls) were analyzed, we found that miR-107 and miR-574-3p were present at significantly higher concentrations in the urine of men with cancer when compared with controls (ANOVA *P*<0.01, Table 3 and Figure 3). Both miRs could identify the presence of prostate cancer from urine samples (concordance indices 0.66–0.74) and appeared more accurate than PCA3 normalised to urinary PSA (concordance index 0.61).

## Discussion

Here, we have used high throughput profiling to identify 12 miRs whose concentration is significantly different (11 miRs whose concentration is significantly greater and 1 miR whose concentration is reduced) in the plasma and serum of patients with prostate cancer, when compared with controls. We confirmed the finding of two of these (miR-574-3p and miR-107) using individual Taqman qRT–PCR assays in two new patient cohorts, using both serum and urine. A total of 16 miRs were found to have significantly altered concentrations in metastatic prostate cancer patients compared with non-metastatic cases, 2 of which (miR-375 and miR-200b) were confirmed by Taqman qRT–PCR assays. It is noted that the patients in our validation cohort had a median PSA of 324.6 compared with a median PSA of 39.2 in our discovery cohort, and this suggests that patients in the validation cohort had a higher burden of disease. Despite this it is noteworthy that we were able to observe changes in particular miR species in two separate cohorts of patients with metastatic prostate cancer compared with those with non-metastatic disease.

Several of these miRs are associated with prostate cancer for the first time to the best of our knowledge, while other miR species, such as miR-141 and miR-331-3p, have been associated with prostate cancer in previous studies (Wang *et al*, 2009). We included an individual Taqman qRT–PCR analysis of miR-141 in our experiments in both the initial plasma cohort and the subsequent serum validation cohort, as miR-141 changes in prostate cancer have been reported in the literature, even though the difference in miR-141 quantity did not reach statistical significance in our initial Exiqon qRT–PCR microarray panel screen. A case–control study of 25 metastatic prostate cancer patients and 25 healthy age-matched male controls demonstrated that the concentration of miR-141 is 46-fold greater in men with metastatic prostate cancer, and measurement of serum levels of miR-141 distinguished patients with metastatic prostate cancer from healthy individuals (Mitchell *et al*, 2008). The results of our study, taken together with others in the literature, suggest that the incorporation of an assay measuring plasma miR-141 concentration within a diagnostic test for metastatic prostate cancer may be clinically useful, particularly within the context of identifying micro-metastatic disease in patients potentially undergoing radical local therapy such as prostatectomy in the presence of a falsely-negative radionuclide bone scan.

A recent combined differential expression and co-expression network analysis of lymphoblastoid cell lines derived from 62 patients with aggressive prostate cancer and 63 patients with non-aggressive prostate cancer identified a panel of 7 differentially expressed miRs associated with aggressive disease. This approach demonstrates the fact that differential expression of particular miRNAs at germline level may dysregulate target hub genes in prostate cancer cells thereby leading to abnormal cell division and proliferation and the development of aggressive prostate cancer. Interestingly, miR-331-3p was one of two miRs predicted to target 3 of the 20 hub genes associated with aggressive prostate cancer (Wang *et al*, 2009). Our study of plasma samples has identified miR-331-3p as being significantly associated with the presence of prostate cancer, suggesting that this miR may be mechanistically important in the development and/or progression of this malignancy.

We identified nine miRs significantly upregulated and one miR downregulated in patients with non-metastatic prostate cancer compared with normal control individuals. Of these, we confirmed the finding with miR-107 using an individual Taqman qRT–PCR assay in both serum and urine. This observation suggests that an individual assay of miR-107 concentration level may be a clinically useful diagnostic test for non-metastatic prostate cancer. Further studies are required to determine if this use of a miR-107 assay using plasma samples may be sensitive and specific enough for use as a single diagnostic test for non-metastatic prostate cancer, followed by prostate biopsy or as part of a panel of measurements including PSA and a subsequent biopsy. The utilisation of assays such as plasma miR-107 measurement may potentially improve the accuracy of conventional prostate cancer detection and case finding when combined with PSA level measurement, although this hypothesis requires formal testing in larger prospective studies.

Our comparison of the miR profile of plasma samples from patients with metastatic prostate cancer compared with non-metastatic cases of this malignancy demonstrated 15 miRs with significantly higher concentration and one miR with significantly lower concentration in association with prostate cancer metastases. The findings in two of these, miR-200b and miR-375, were confirmed using Taqman qRT–PCR assays. MicroRNA-200b and miR-375 have recently been shown to be increased in the serum of patients with metastatic prostate cancer compared with patients with localised disease (Brase *et al*, 2011), hence these miRs might represent useful markers of micro-metastases in order to aid appropriate selection of patients with organ-confined prostate cancer for invasive therapy such as radical prostatectomy. Although serum PSA measurement is extremely valuable in the diagnosis of patients with established metastases, it is less accurate in the identification of patients with occult micro-metastases, potentially leading to inappropriate treatment choices such as radical surgery. Our verification of the use of miR-141 and miR-375 as markers of metastatic prostate cancer using independent exosomal and microvesicle samples corroborates evidence from other studies implicating miR-141 and miR-375 as useful candidate markers of a metastatic prostate cancer signature (Brase *et al*, 2011). It is possible that assays measuring the concentration of miR-141 and/or miR-375 may find clinical utility as biomarkers incorporated into a suitable blood test for the differential diagnosis of metastatic prostate cancer. This warrants further investigation in a larger prospective study, particularly in the context of detecting micro-metastases, which are too small to be detected with conventional investigations such as radionuclide bone scans or magnetic resonance imaging of bone marrow. A miR-based test may possibly enable clinicians to distinguish more accurately indolent or non-metastatic prostate cancer from aggressive disease with metastatic potential. This distinction may facilitate the selection of appropriate curative therapeutic options for patients with potentially aggressive disease, enabling other patients with more indolent disease to avoid complications and side effects associated with over-treatment of prostate cancer.

In summary, we have demonstrated that changes in miR concentration may be detected in plasma and serum samples and may be useful as an aid in the diagnosis of prostate cancer. We have also identified a miR signature of localised or metastatic prostate cancer, which may be useful in the identification of occult micro-metastases. Blood-based assays of particular miR concentrations may represent novel and clinically beneficial tests for different aspects of prostate cancer management and warrant further investigation in large prospective patient cohorts.

## Figures and Tables

**Table 1:** Characteristics of ProMPT (plasma and urine sample) and University of Washington (serum sample) patients

	**ProMPT**					**University of Washington**	
	**Plasma**		**Urine**			**Serum**	
	**Normal controls**	**Prostate cancer**	**Normal controls**	**Localised prostate cancer**	**Advanced prostate cancer**	**Non-recurrent**	**Metastatic**
Mean age (years)	63	70	69	69	73	59.9	69.6
Mean PSA ng ml^−1^ (s.d.)	5.69 (5.76)	86.4 (201.8)	4.1 (2.3)	9.6 (7.9)	63.6 (266.4)	< 4.0	603 (794)
Median PSA ng ml^−1^ (IQR)	4.0 (2.1–7.8)	19.15 (7.9–44.1)	3.2 (1.7–6.9)	8.4 (1.3–22.3)	39.2 (19.2–530)	N/A	324.6 (152.1–565)
Gleason score^a^ ⩽6	—	33	—	52	12	46	4
7	—	19	—	14	18	25	5
⩾8	—	20	—	4	18	1	22
pT stage pTx	—	4	—	—	—	1	16
PT1/2	—	35	—	70	—	64	—
PT3/4	—	39	—	—	48	7	—
LN stage Nx	—	73	—	—	—	—	—
N0	—	3	—	—	—	—	—
N1	—	2	—	—	—	1	—
M stage Mx	—	12	—	—	—	72	0
M0	—	51	—	—	—	—	—
M1	—	15	—	—	—	—	47
ADT Yes	—	—	—	7	37	0	33
No	—	—	—	63	11	72	14
Total (*n*)	28	78^a^	17	70	48	72	47

Abbreviations: ProMPT=Prostate Cancer Mechanisms of Progression and Treatment; PSA=prostate-specific antigen; IQR=interquartile range.

a Gleason Score data available for 72 of 78 ProMPT plasma samples.

**Table 2A:** Analysis of all prostate cancer cases versus normal control individuals

	**Normal control individuals**		**Prostate cancer**			
**MiR**	**Normalised CT value**	**s.d.**	**Normalised CT value**	**s.d.**	**Fold change**	**Corrected *P***-**value**
107	−5.10	5.20	−1.50	4.20	11.26	0.034
130b	−2.00	2.60	0.19	3.50	4.72	0.034
141	−0.73	0.99	1.30	3.50	4.29	0.034
181a-2^*^	1.10	3.50	−0.40	1.10	−2.69	0.034
2110	−1.90	3.20	0.69	4.20	6.13	0.035
301a	−1.90	3.10	0.57	4.00	5.59	0.035
326	−2.20	2.00	0.20	3.90	5.28	0.034
331-3p	−2.00	3.30	0.43	4.10	5.39	0.043
432	−0.70	1.00	1.10	3.20	3.5	0.035
484	−1.90	3.10	−0.37	2.30	2.87	0.47
574-3p	−2.20	3.40	−0.38	1.90	3.48	0.034
625^*^	−0.68	1.00	1.20	3.40	3.76	0.035

**Table 2B:** Analysis of localised prostate cancer cases versus normal control individuals

	**Normal control individuals**		**Localised prostate cancers**			
**MiR**	**Normalised CT value**	**s.d.**	**Normalised CT value**	**s.d.**	**Fold change**	**Corrected *P*-value**
107	−5.1	5.2	−1.4	3.9	11.26	0.034
141	−0.73	1	0.96	3.2	4.29	0.034
181a-2^*^	1.1	3.5	−0.4	1.2	−2.69	0.034
2110	−1.9	3.2	0.66	4.2	6.13	0.035
301a	−1.9	3.1	0.5	4.0	5.59	0.035
326	−2.2	2	0.49	3.9	5.28	0.034
432	−0.7	1	1.4	3.4	3.5	0.035
484	−1.89	3.1	−0.34	2.3	2.87	0.47
574-3p	−2.17	3.4	−0.44	1.9	3.48	0.034
625^*^	−0.68	1	1.33	3.5	3.76	0.035

**Table 2C:** Analysis of metastatic prostate cancer cases versus localised prostate cancer cases

	**Localised prostate cancer**		**Metastatic prostate cancer**			
**MiR**	**Normalised CT value**	**s.d.**	**Normalised CT value**	**s.d.**	**Fold change**	**Corrected *P*-value**
582-3p	−0.52	0.87	0.57	2.10	2.51	0.001
20a^*^	−0.46	0.87	0.61	2.70	3.62	0.002
375	0.83	3.50	4.21	1.40	10.71	0.003
200b	−0.21	1.70	1.60	3.60	3.90	0.003
379	−0.43	0.87	0.38	2.00	2.10	0.005
572	0.63	3.70	−2.70	1.40	−7.39	0.005
513a-5p	−0.44	0.87	0.46	2.36	2.23	0.005
577	−0.15	2.38	1.64	3.96	5.90	0.005
23a^*^	−0.46	0.87	0.49	2.53	2.30	0.005
1236	−0.43	0.87	0.73	3.23	2.63	0.005
609	−0.46	0.87	0.50	2.68	2.31	0.006
17^*^	0.29	2.40	2.40	4.12	4.80	0.006
619	−0.28	1.41	0.80	2.52	3.37	0.008
624^*^	0.03	2.70	1.91	4.16	6.09	0.009
198	−0.49	0.87	0.34	2.40	2.12	0.009
130b	−0.47	3.20	2.18	3.60	6.12	0.007

Tables 2A, B and C: Exiqon qRT–PCR microarray panel-detected miR concentration changes in plasma-derived circulating microvesicles associated with aspects of prostate cancer. Eleven miRs were present in significantly greater amounts in prostate cancer patients compared with normal control individuals (no prostate cancer) and nine of these were increased in patients with localised prostate cancer (*P*<0.05 unpaired *t*-test). In both cases miR-181a-2^*^ was present in significantly less concentration. A total of 16 miRs were found to be present at different concentrations in prostate cancer patients with metastases compared with those with non-metastatic disease, 15 showed a greater concentration, while the concentration of miR-572 was significantly decreased (*P*<0.01 unpaired *t*-test).

**Table 3:** Quantification of selected microRNAs in urinary cells from patients with prostate cancer and controls

		**ΔCt**		**95% CI**		**ANOVA**
**Sample**	***N***	**Mean**	**s.d.**	**Lower**	**Upper**	***P*-value**
*miR-107*						
Benign	17	10.90	5.18	8.24	13.56	0.001
Prostate cancer	113	7.93	2.86	7.40	8.47	
ND	5					
						
*miR-574-3p*						
Benign	17	3.19	2.04	2.14	4.24	0.012
Prostate cancer	115	1.82	2.08	1.44	2.21	
ND	3					
						
*miR-375*						
Benign	17	2.65	3.54	0.83	4.48	0.376
Prostate cancer	115	1.84	3.53	1.19	2.49	
ND	3					
*miR-200b*						
Benign	17	2.35	2.12	1.26	3.44	0.533
Prostate cancer	116	1.91	2.83	1.39	2.43	
ND	2					
						
*miR-141*						
Benign	17	4.94	2.12	3.85	6.03	0.752
Prostate cancer	115	4.73	2.62	4.24	5.21	
ND	3					

Abbreviation: ND=not detectable.

## References

[bib1] Bartel DP (2009) MicroRNAs: target recognition and regulatory functions. Cell 136: 215–233 19167326 10.1016/j.cell.2009.01.002 PMC3794896

[bib2] Brase JC, Johannes M, Schlomm T, Falth M, Haese A, Steuber T, Beissbarth T, Kuner R, Sultmann H (2011) Circulating miRNAs are correlated with tumor progression in prostate cancer. Int J Cancer 128: 608–616 20473869 10.1002/ijc.25376

[bib3] Catto JW, Alcaraz A, Bjartell AS, De Vere White R, Evans CP, Fussel S, Hamdy FC, Kallioniemi O, Mengual L, Schlomm T, Visakorpi T (2011) MicroRNA in prostate, bladder, and kidney cancer: a systematic review. Eur Urol 59: 671–681 21296484 10.1016/j.eururo.2011.01.044

[bib4] Catto JW, Miah S, Owen HC, Bryant H, Myers K, Dudziec E, Larre S, Milo M, Rehman I, Rosario DJ, Di Martino E, Knowles MA, Meuth M, Harris AL, Hamdy FC (2009) Distinct microRNA alterations characterize high- and low-grade bladder cancer. Cancer Res 69: 8472–8481 19843843 10.1158/0008-5472.CAN-09-0744 PMC2871298

[bib5] Ciatto S, Zappa M, Bonardi R, Gervasi G (2000) Prostate cancer screening: the problem of overdiagnosis and lessons to be learned from breast cancer screening. Eur J Cancer 36: 1347–1350 10899646 10.1016/s0959-8049(00)00119-2

[bib6] Croce CM (2009) Causes and consequences of microRNA dysregulation in cancer. Nat Rev Genet 10: 704–714 19763153 10.1038/nrg2634 PMC3467096

[bib7] Dall’Era MA, Cooperberg MR, Chan JM, Davies BJ, Albertsen PC, Klotz LH, Warlick CA, Holmberg L, Bailey Jr DE., Wallace ME, Kantoff PW, Carroll PR (2008) Active surveillance for early-stage prostate cancer: review of the current literature. Cancer 112: 1650–1659 18306379 10.1002/cncr.23373

[bib8] Hessels D, Klein Gunnewiek JM, van Oort I, Karthaus HF, van Leenders GJ, van Balken B, Kiemeney LA, Witjes JA, Schalken JA (2003) DD3(PCA3)-based molecular urine analysis for the diagnosis of prostate cancer. Eur Urol 44: 8–15; discussion 15-6 12814669 10.1016/s0302-2838(03)00201-x

[bib9] Jemal A, Siegel R, Xu J, Ward E (2010) Cancer statistics, 2010. CA Cancer J Clin 60: 277–300 20610543 10.3322/caac.20073

[bib10] Mitchell PS, Parkin RK, Kroh EM, Fritz BR, Wyman SK, Pogosova-Agadjanyan EL, Peterson A, Noteboom J, O’Briant KC, Allen A, Lin DW, Urban N, Drescher CW, Knudsen BS, Stirewalt DL, Gentleman R, Vessella RL, Nelson PS, Martin DB, Tewari M (2008) Circulating microRNAs as stable blood-based markers for cancer detection. Proc Natl Acad Sci USA 105: 10513–10518 18663219 10.1073/pnas.0804549105 PMC2492472

[bib11] Rajarubendra N, Bolton D, Lawrentschuk N (2010) Diagnosis of bone metastases in urological malignancies – an update. Urology 76: 782–790 20346492 10.1016/j.urology.2009.12.050

[bib12] Slawski M, Daumer M, Boulesteix AL (2008) CMA: a comprehensive bioconductor package for supervised classification with high dimensional data. BMC Bioinform 9: 439 10.1186/1471-2105-9-439 PMC2646186 18925941

[bib13] Thompson IM, Goodman PJ, Tangen CM, Lucia MS, Miller GJ, Ford LG, Lieber MM, Cespedes RD, Atkins JN, Lippman SM, Carlin SM, Ryan A, Szczepanek CM, Crowley JJ, Coltman Jr CA (2003) The influence of finasteride on the development of prostate cancer. N Engl J Med 349: 215–224 12824459 10.1056/NEJMoa030660

[bib14] Wang L, Tang H, Thayanithy V, Subramanian S, Oberg AL, Cunningham JM, Cerhan JR, Steer CJ, Thibodeau SN (2009) Gene networks and microRNAs implicated in aggressive prostate cancer. Cancer Res 69: 9490–9497 19996289 10.1158/0008-5472.CAN-09-2183 PMC2795036

[bib15] Weber JA, Baxter DH, Zhang S, Huang DY, Huang KH, Lee MJ, Galas DJ, Wang K (2010) The microRNA spectrum in 12 body fluids. Clin Chem 56: 1733–1741 20847327 10.1373/clinchem.2010.147405 PMC4846276

